# The mitochondrial genome of the oribatid mite *Paraleius leontonychus*: new insights into tRNA evolution and phylogenetic relationships in acariform mites

**DOI:** 10.1038/s41598-018-25981-w

**Published:** 2018-05-15

**Authors:** Sylvia Schäffer, Stephan Koblmüller, Ingeborg Klymiuk, Gerhard G. Thallinger

**Affiliations:** 1 Institute of Biology, University of Graz, Universitätsplatz 2, 8010 Graz, Austria; 2 Core Facility Molecular Biology, Center for Medical Research, Medical University of Graz, Graz, Stiftingtalstraße 24, 8010 Graz, Austria; 3 Institute of Computational Biotechnology, Graz University of Technology, Petersgasse 14, 8010 Graz, Austria; 4 OMICS Center Graz, BioTechMed Graz, Stiftingtalstraße 24, 8010 Graz, Austria

## Abstract

Bilaterian mitochondrial (mt) genomes are circular molecules that typically contain 37 genes. To date, only a single complete mitogenome sequence is available for the species-rich sarcoptiform mite order Oribatida. We sequenced the mitogenome of *Paraleius leontonychus*, another species of this suborder. It is 14,186 bp long and contains 35 genes, including only 20 tRNAs, lacking *tRNA*^*Gly*^ and *tRNA*^*Tyr*^. Re-annotation of the mitogenome of *Steganacarus magnus* increased the number of mt tRNAs for this species to 12. As typical for acariform mites, many tRNAs are highly truncated in both oribatid species. The total number of tRNAs and the number of tRNAs with a complete cloverleaf-like structure in *P. leontonychus*, however, clearly exceeds the numbers previously reported for Sarcoptiformes. This indicates, contrary to what has been previously assumed, that reduction of tRNAs is not a general characteristic for sarcoptiform mites. Compared to other Sarcoptiformes, the two oribatid species have the least rearranged mt genome with respect to the pattern observed in *Limulus polyphemus*, a basal arachnid species. Phylogenetic analysis of the newly sequenced mt genome and previously published data on other acariform mites confirms paraphyly of the Oribatida and an origin of the Astigmata within the Oribatida.

## Introduction

Studies on the description of complete mitochondrial (mt) genomes have accumulated in recent years, as, with the advent of high-throughput sequencing methods, it has become much easier to quickly obtain accurate mitogenome assemblies from any target species of choice^1–4^. Moreover, free user-friendly software or online tools are available, providing easy and fast automated gene annotation, which in turn enables generation of a draft mitogenome in a matter of weeks^4–6^.

Bilaterian animals have a circular mitogenome, usually 13–17 kb in size, with a relatively conserved gene content, usually comprising 37 genes. These are 13 protein coding genes (PCGs), 2 ribosomal RNA (rRNA) genes, and 22 different transfer (tRNA) genes. In addition, animals also have an A + T-rich control region^7,8^. Interspecific length variation of mitogenomes is usually due to length variation in the control region and/or presence/absence of particular tRNA genes, but also due to length differences in the PCGs^9^. Complete mitogenomes represent important molecular resources not only for phylogenetic, phylogeographic and population genetic studies^10–12^, but are also interesting and relevant with respect to gene order evolution^13,14^ or adaption to novel environments^15,16^. Although there are several bioinformatics tools available, identifying mt-tRNAs is not always straight forward, in particular in case of unknown codon/anticodon rules, post-transcriptional modifications, deviations from the standard genetic code or unconventional secondary structures^17,18^. Additionally, it is well known that the presence of the complete 22 tRNAs is not universal as there are several mitogenomes (also in metazoans) lacking one to all tRNA genes^18–20^.

In general, more than 90% of the metazoan mt-tRNAs are inferred to possess the conventional four-armed cloverleaf secondary structure. There is at least one well-known exception, the D–arm lacking *tRNA*^Ser (AGN/Y)^, a feature shared among nearly all Metazoa^21,22^. So far, there are several known mitogenomes whose encoded tRNAs are non-canonical which means that they have either reduced D- or T-arms (truncated tRNAs), or even lack these two elements at all (“armless” tRNA), resulting in very short encoding genes. An extreme case of armless tRNAs has been found in the mitochondria of the nematode class Enoplea, where encoded transcripts have a length of only 42 nucleotides (nts)^22^, representing the world’s smallest tRNA (typical tRNA length is 70–100 nts). Biological activity of these extremely short tRNAs was demonstrated by Wende *et al*.^17^ by verification of *in vitro* transcription and 3′- and 5′-processing of several mt-tRNAs of the mermithid *Romanomermis culicivorax*. Beside nematodes, evidence for truncated tRNAs has been found in several other groups, but most frequently in arthropods^22,23^ and in particular in mites. While the majority of species from the superorder Parasitiformes do not have more than two truncated tRNAs^24–26^, reduction of tRNA-D- and/or T-arms has been shown in all published mitogenomes of the second superorder Acariformes^22,27,28^ known so far. In general, only the *tRNA*^*Lys*^ shows the typical cloverleaf structure in all known acariform species, except for the oribatid mite *Steganacarus magnus*^29^. Three further tRNAs lack the T-arm in all Acariformes, representing a potential ancestral feature, while all remaining 18 tRNAs vary in their secondary structure among the same mite species^28^. In contrast to the aberrant secondary structures, losing a tRNA is not typical for arthropods^30^. However, there are a few documented examples, as e.g. in the Chinese scorpion *Mesobuthus martensii*^31^, in some isopods^32,33^ and also in mites. Whereas all published parasitiform mitogenomes have the full set of tRNA genes, four species in the Acariformes are known to have a reduced number of mt-tRNA, namely *S. magnus*^29^, *Sarcoptes scabiei*^34^ and two *Tyrophagus* species^35^. While the mt genomes of *S. scabiei* and *Tyrophagus* spp. lack two, respectively, three tRNAs, *S. magnus* lost 16 tRNAs^29^.

In general, acariform mites typically show high levels of mt gene rearrangement, loss of tRNAs and unconventional secondary structures of tRNAs, which makes them an ideal model system for studying gene order and tRNA evolution. Mite systematics, in general, is complex and controversial and also for acariform mites there are several classification schemes present in the literature^36–39^. In the current study we refer to the classification scheme of Lindquist *et al*.^37^ who divide the Acariformes into the two orders Trombidiformes and Sarcoptiformes. These two orders include several suborders wherein families are subdivided into various cohorts and/or supercohorts.

The species investigated here, *Paraleius leontonychus*, is a very unusual member of the sarcoptiform suborder Oribatida. Its special feature is undoubtedly not only the typical arboreal life-style; but especially its unusual form of dispersal. *Paraleius leontonychus* is one of few acariform mite species that use other arthropods, more precisely bark beetles of the subfamily Scolytinae, as host organisms for transport^40,41^. As a specific morphological adaptation for this so-called phoretic behavior, this mite species exhibits a strong hook-like claw on each tarsus with which it adheres to its host^42^.

In the present study we sequenced and analyzed the complete mitogenome of *P. leontonychus* (as part of an ongoing whole genome assembly and annotation project) to investigate its impact on the evolution of tRNAs within the Acariformes as well as on the phylogeny of the Sarcoptiformes. We compared the new mitogenome with those of other closely related species and performed gene rearrangement analyses relative to *Limulus polyphemus*, as the hypothetical ancestor of arachnids. The mt genome of *S. magnus* was originally described by Domes *et al*.^29^ to exhibit an unexpectedly great loss of tRNAs (only 6 of 22 present). In a later study, Klimov & OConnor^27^ provided an improved tRNA prediction in the house dust mite *Dermatophagoides farinae* including tRNAs of *S. magnus* for a comparison. Like Domes *et al*.^29^, Klimov and OConnor used tRNAscan-SE^43^ and ARWEN^44^ to predict tRNAs and infer their secondary structure. In contrast to Domes *et al*.^29^, the minimum free energy (MFE) of the constrained and unconstrained secondary structure was additionally calculated to select the most probable of alternative predicted structures^27^. Klimov and OConnor^27^ identified another two tRNAs and re-annotated three of the previously described ones based on manual sequence annotation and MFE calculations. With this background, we decided to re-annotate the tRNAs in the *S. magnus* mitogenome once more using the same programs and prediction methods as for *P. leontonychus* and compared our results with those from the former studies^27^. Beyond that, *S. magnus* is of particular interest as it belongs to the oribatid supercohort Mixonomatides, which represents a phylogenetically more basal group compared to species of the supercohort Desmonomatides^45^, which includes *P. leontonychus*. Considering the presumed close relationship of *S. magnus* and *P. leontonychus*, we expect a similarly extensive loss of tRNA genes in *P. leontonychus*.

## Results

**Figure 1:**
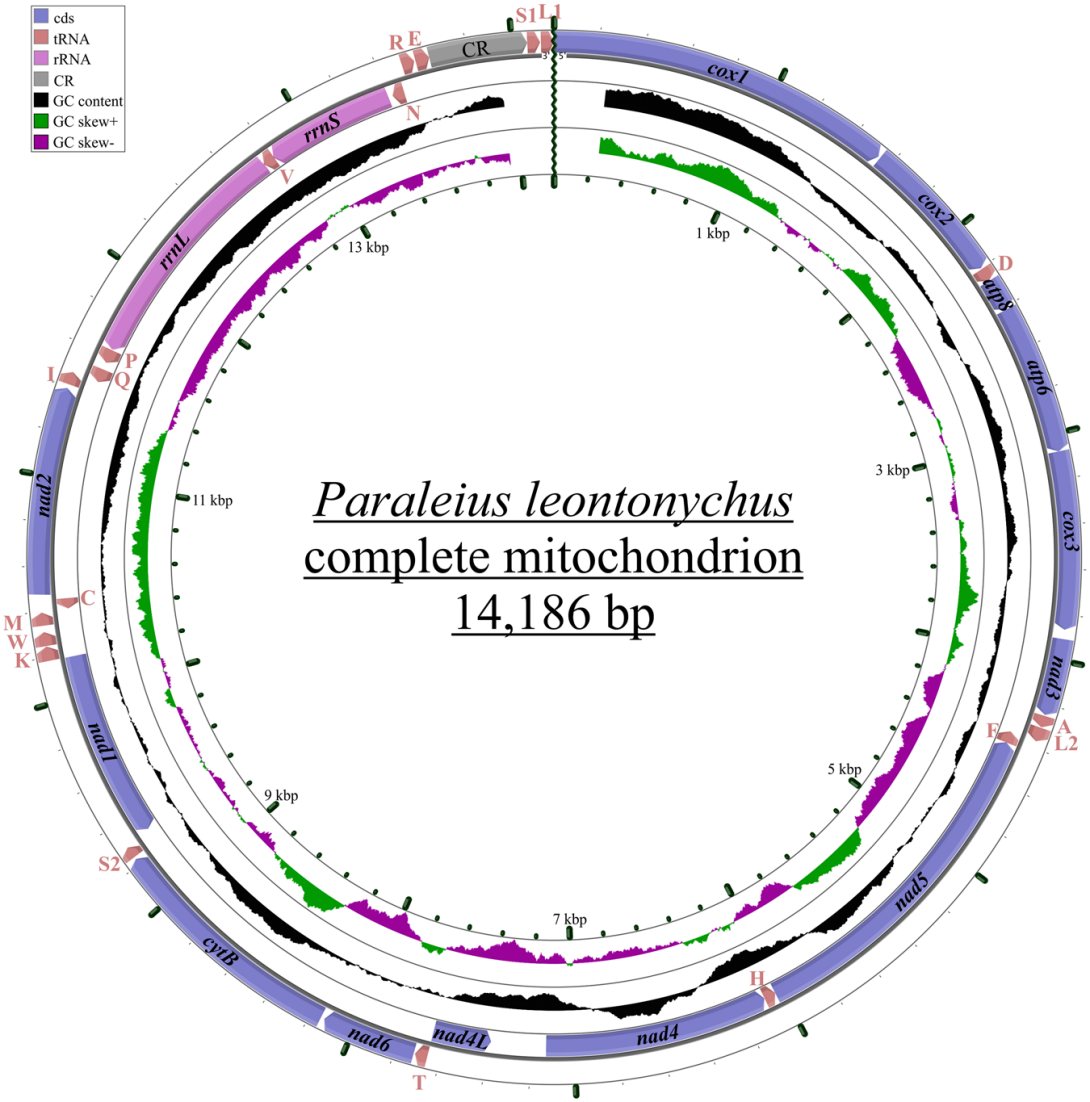
Mitochondrial genome of *P. leontonychus*. Genes transcribed on the leading (+)-strand are on the outside of the circles, those on the lagging (−)-strand on the inside of the circles. Color codes for the genes are given in the box; tRNAs are abbreviated by the one-letter code for the corresponding amino acid. All abbreviations are the same as in Table 1.

The mitogenome of *P. leontonychus* is a closed circular DNA molecule that is 14,186 nts long and encodes for 35 genes, 13 PCGs, two ribosomal RNAs and 20 tRNAs (Fig. 1, Table 1). While PCGs and tRNAs are located on both strands, the two rRNAs are encoded on the (−)-strand. Ten of the 13 PCGs start with the mt start codons ATA, ATC or ATT, while *nad4* and *nad1* use TTG and *nad6* the start codon GTG. The stop codons are either TAA or TAG, and incomplete stop codons, T or TA, are present in those PCGs that overlap with other coding genes or tRNAs. The nucleotide composition of the leading (+)-strand is A = 38.8%, C = 22.0%, G = 13.3% and T = 25.8%, resulting in a positive AT-skew (0.201) and a negative GC-skew (−0.245).

**Table 1:** Mitochondrial genome organization of *Paraleius leontonychus*.

Gene	Product/Description	Start	End	Strand	Length	Gap	Startcodon	Stopcodon
cox1	cytochrome c oxidase subunit I	1	1536	+	1536	9	ATA	TAA
cox2	cytochrome c oxidase subunit II	1546	2202	+	657	5	ATA	TAA
trnD	tRNA-Asp(gtc)	2208	2267	+	60	1		
atp8	ATP synthase F0 subunit 8	2269	2413	+	145	1	ATC	T(AA)
atp6	ATP synthase F0 subunit 6	2415	3079	+	665	0	ATA	TA(A)
cox3	cytochrome c oxidase subunit III	3080	3865	+	786	41	ATA	TAA
nad3	NADH dehydrogenase subunit 3	3907	4248	+	342	13	ATT	TAA
trnA	tRNA-Ala(tgc)	4262	4306	+	45	1		
trnL2	tRNA-Leu(taa)	4308	4370	+	63	−1		
trnF	tRNA-Phe(gaa)	4370	4424	−	55	0		
nad5	NADH dehydrogenase subunit 5	4425	6033	−	1609	0	ATT	T(AA)
trnH	tRNA-His(gtg)	6034	6088	−	55	1		
nad4	NADH dehydrogenase subunit 4	6090	7130	−	1041	255	TTG	TAA
nad4L	NADH dehydrogenase subunit 4L	7386	7655	−	270	0	ATA	TAA
trnT	tRNA-Thr(tgt)	7656	7710	+	55	0		
nad6	NADH dehydrogenase subunit 6	7711	8136	+	426	1	GTG	TAA
cytB	cytochrome b	8138	9232	+	1095	1	ATA	TAA
trnS2	tRNA-Ser(tga)	9234	9287	+	54	−2		
nad1	NADH dehydrogenase subunit 1	9286	10182	−	897	1	TTG	TAG
trnK	tRNA-Lys(ttt)	10184	10247	+	64	2		
trnW	tRNA-Trp(tca)	10250	10311	+	62	28		
trnM	tRNA-Met(cat)	10340	10397	+	58	−5		
trnC	tRNA-Cys(gca)	10393	10463	−	71	1		
nad2	NADH dehydrogenase subunit 2	10465	11406	+	942	7	ATA	TAA
trnI	tRNA-Ile(gat)	11414	11470	+	57	−2		
trnQ	tRNA-Gln(ttg)	11469	11531	−	63	34		
trnP	tRNA-Pro(tgg)	11566	11629	−	64	13		
rrnL	large ribosomal RNA	11643	12751	−	1109	0		
trnV	tRNA-Val(tac)	12752	12798	−	47	−4		
rrnS	small ribosomal RNA	12795	13477	−	683	−2		
trnN	tRNA-Asn(gtt)	13476	13531	−	56	−14		
trnR	tRNA-Arg(tcg)	13518	13563	+	46	8		
trnE	tRNA-Glu(ttc)	13572	13633	+	62	0		
CR	control region	13634	14068	+	435	0		
trnS1	tRNA-Ser(gct)	14069	14125	+	57	3		
trnL1	tRNA-Leu(tag)	14129	3	+	61	−3		

A control region (CR) with a length of 435 nts was predicted, which is comparable to annotated CR sequences of other published sarcoptiform mitogenomes (except for *S. magnus* [1019 nts] and *Tyrophagus longior* [50 nts]). The AT content of the CR is 59.3%, which is considerable lower than for the other Sarcoptiformes (69.0–91.6%). Together with the lack of longer A and T stretches this leads to smaller stemloop structures compared to the related species. The position and number of the stemloops do not seem to be conserved.

**Figure 2:**
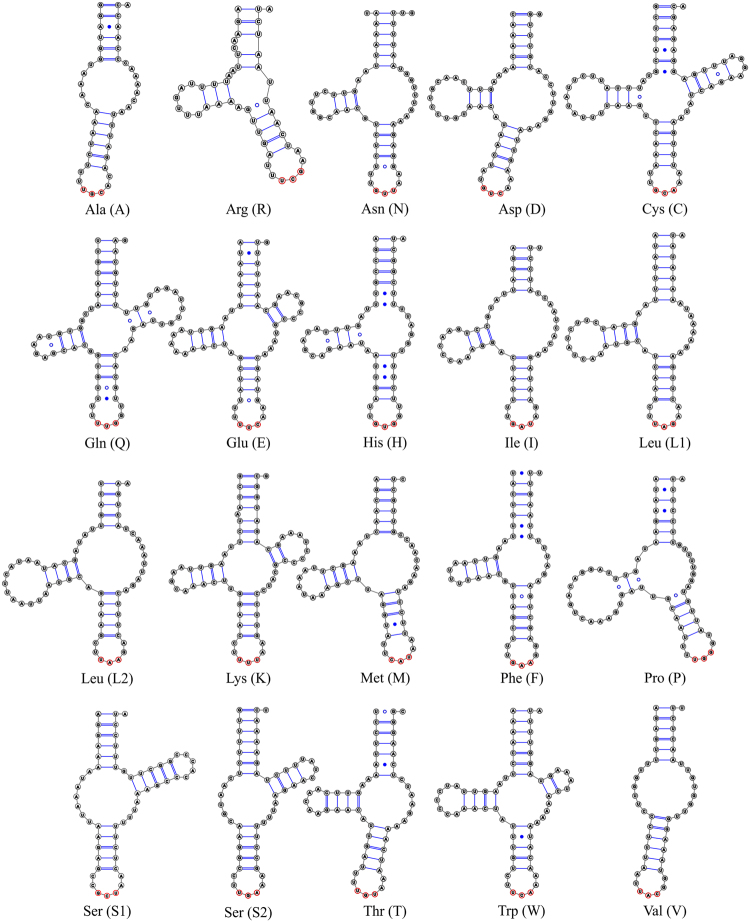
Predicted secondary structures of the 20 mt tRNAs of *P. leontonychus*.

For *P. leontonychus*, eighteen tRNAs were detected by at least one of the programs used (Supplementary Table S1, Supplementary Fig. S1), while *tRNA*^*Ala*^ and *tRNA*^*Val*^ were identified manually only based on the anticodon sequences and the conserved secondary structure. *tRNA*^*Gly*^ and *tRNA*^*Tyr*^ could not be identified. In general, most of the tRNAs are short and do not show the typical clover-leaf secondary structure. They are highly truncated and miss either the D-, T-arm or both arms (=“armless” tRNA). About half of them have mismatched base pairs and/or truncated acceptor stems (with less than seven paired bases, e.g. *tRNA*^*Ala*^, *tRNA*^*Leu2*^, *tRNA*^*Met*^, *tRNA*^*Ser1*^, *tRNA*^*Val*^; Fig. 2). Furthermore, we observed tRNA genes overlapping with other tRNAs (*tRNA*^*Leu2*^/*tRNA*^*Phe*^, *tRNA*^*Met*^/*tRNA*^*Cys*^, and *tRNA*^*Ile*^/*tRNA*^*Gln*^), but no overlap between tRNAs and PCGs. The annotated sequence has been deposited in the European Nucleotide Archive under the accession number LT984407.

**Figure 3:**
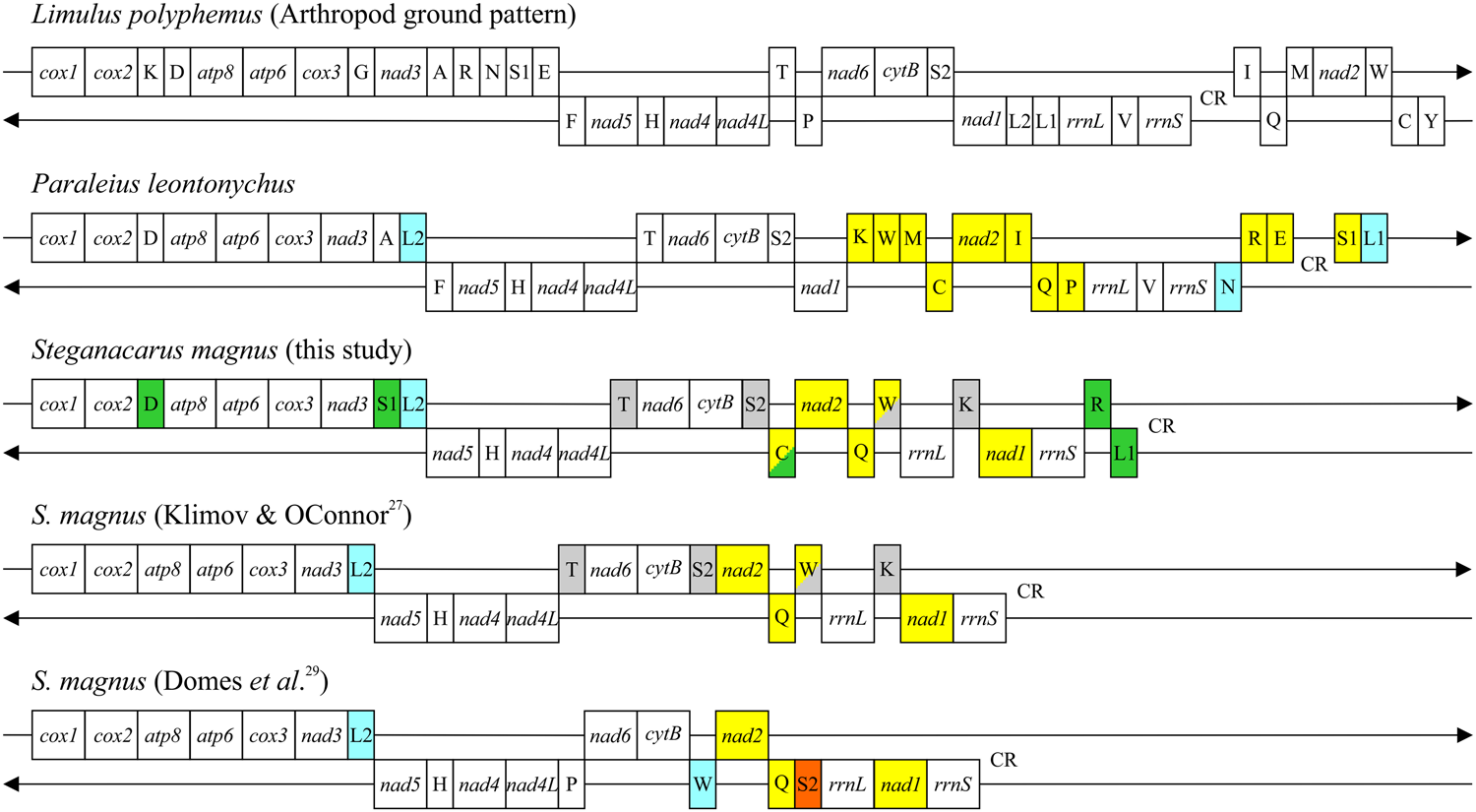
Mitochondrial gene arrangements of the two oribatid species, *P. leontonychus* and *S. magnus*, compared to *L. polyphemus*, representing the arthropod ground pattern. For *S. magnus*, mitogenome orders obtained from different annotations are shown. All abbreviations are the same as in Fig. 1 and Table 1. Arrow pointing to the right represents the (+)-strand and arrow to the left the (−)-strand. Genes are drawn in their original order; intergenic distances are not included and sizes of genes are not true to scale. Orange box represents a gene with a different location relative to *L. polyphemus*; yellow boxes indicate genes that have different positions and ice blue boxes genes that are different in terms of both position and strand associations. Green boxes highlight the newly predicted tRNAs for *S. magnus*, grey boxes indicate congruencies between the re-annotation of Klimov and OConnor^27^, and this study.

The re-annotation of the tRNAs of the *S. magnus* mitogenome predicted 16 novel tRNAs (*tRNA*^*Asp*^*, tRNA*^*Met*^*, tRNA*^*Ser1*^*, tRNA*^*Phe*^*, tRNA*^*Thr*^*, tRNA*^*Ser2*^*, tRNA*^*Cys*^*, tRNA*^*Gln*^*, tRNA*^*Tyr*^*, tRNA*^*Trp*^*, tRNA*^*Glu*^*, tRNA*^*Gly*^*, tRNA*^*Lys*^*, tRNA*^*Ile*^*, tRNA*^*Arg*^*, and tRNA*^*Leu1*^). Filtering the novel tRNAs based on the constrained MFE values left nine reliable predictions (*tRNA*^*Asp*^*, tRNA*^*Ser1*^*, tRNA*^*Thr*^*, tRNA*^*Ser2*^*, tRNA*^*Cys*^*, tRNA*^*Trp*^*, tRNA*^*Lys*^*, tRNA*^*Arg*^*, and tRNA*^*Leu1*^), including three changed tRNA assignments (*tRNA*^*Pro*^->*tRNA*^*Thr*^, *tRNA*^*Trp*^->*tRNA*^*Ser2*^, *tRNA*^*Ser2*^->*tRNA*^*Trp*^: Supplementary Table S2, Supplementary Fig. S2). Together with the three correctly annotated tRNAs in Domes *et al*.^29^, this lead to a new mitogenome arrangement of *S. magnus* with a total of 12 tRNAs (Fig. 3).

**Figure 4:**
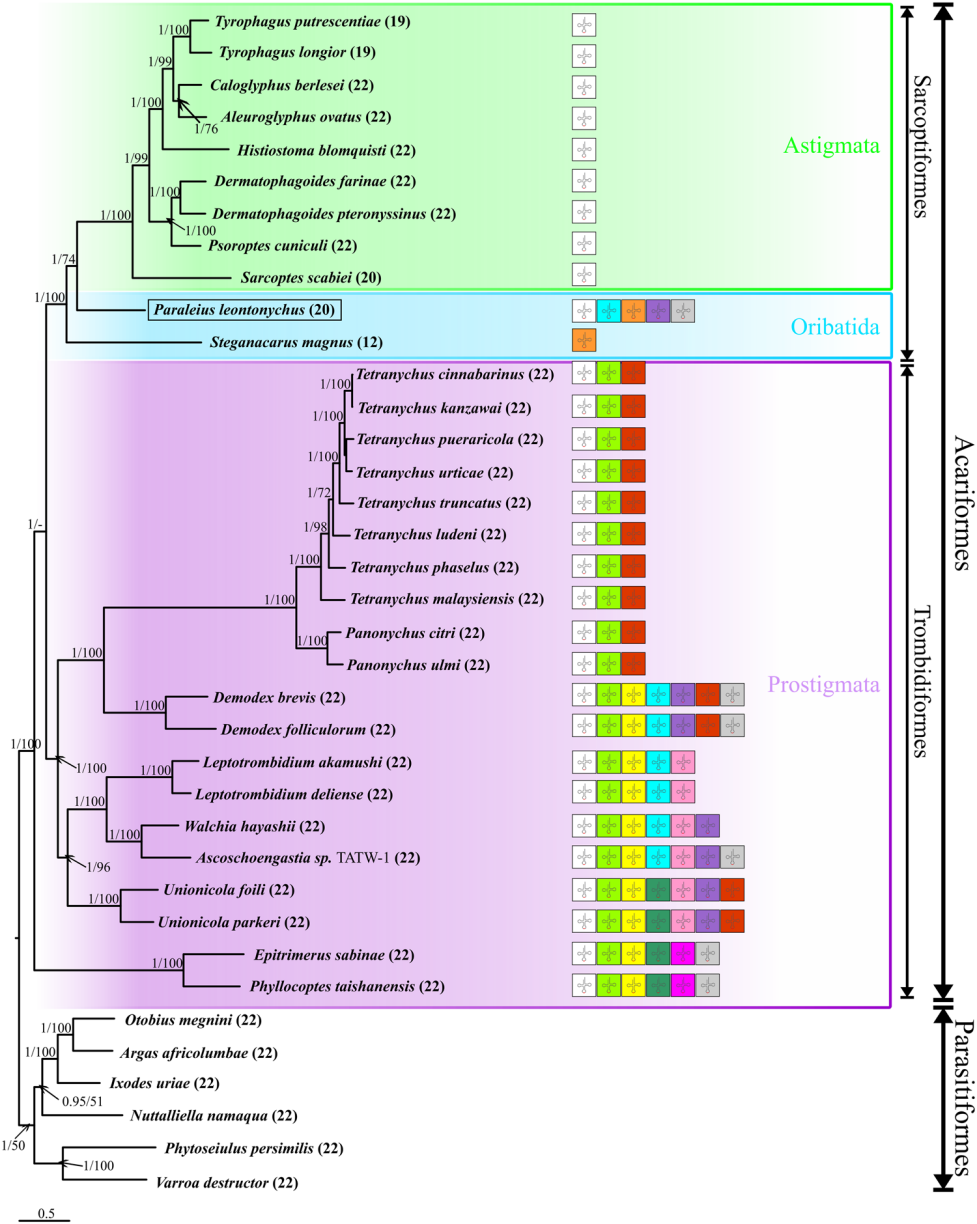
Phylogenetic relationships among 37 mite taxa inferred from Bayesian Inference analyses of nucleotide sequences of PGCs. Numbers at nodes indicate posterior probabilities ML bootstrap values, respectively. Numbers in parentheses following the species names refer to the total number of tRNAs found in that species. For the acariform taxa, tRNAs with typical clover-leaf secondary structure are shown in different colors (C in orange, D in pink, E in ice-blue, G in rose, K in white, L_1_ in dark green, L_2_ in light green, M in yellow, N in red, Q in violet and W in light grey).

The phylogenetic reconstructions based on Maximum likelihood (ML) and Bayesian Inference (BI) analyses revealed identical topologies for both datasets, nucleotide (ND) and amino acid (AAD) sequences of the PCGs. Almost all nodes were statistically well supported by high bootstrap values and high BI posterior probabilities (Fig. 4 and Supplementary Fig. S3). All analyses unambiguously supported the monophyly of the two superorders (Acariformes and Parasitiformes) and the orders Sarcoptiformes, Ixodida and Mesostigmata. The new sequence of *P. leontonychus* was placed as sister group of the Astigmata, rendering the Oribatida paraphyletic. In addition, the order Trombidiformes was recovered as paraphyletic due to the separate placement of the two eriophyoid species, *Epitrimerus sabinae* and *Phyllocoptes taishanensis* at the base of the Acariformes.

**Figure 5:**
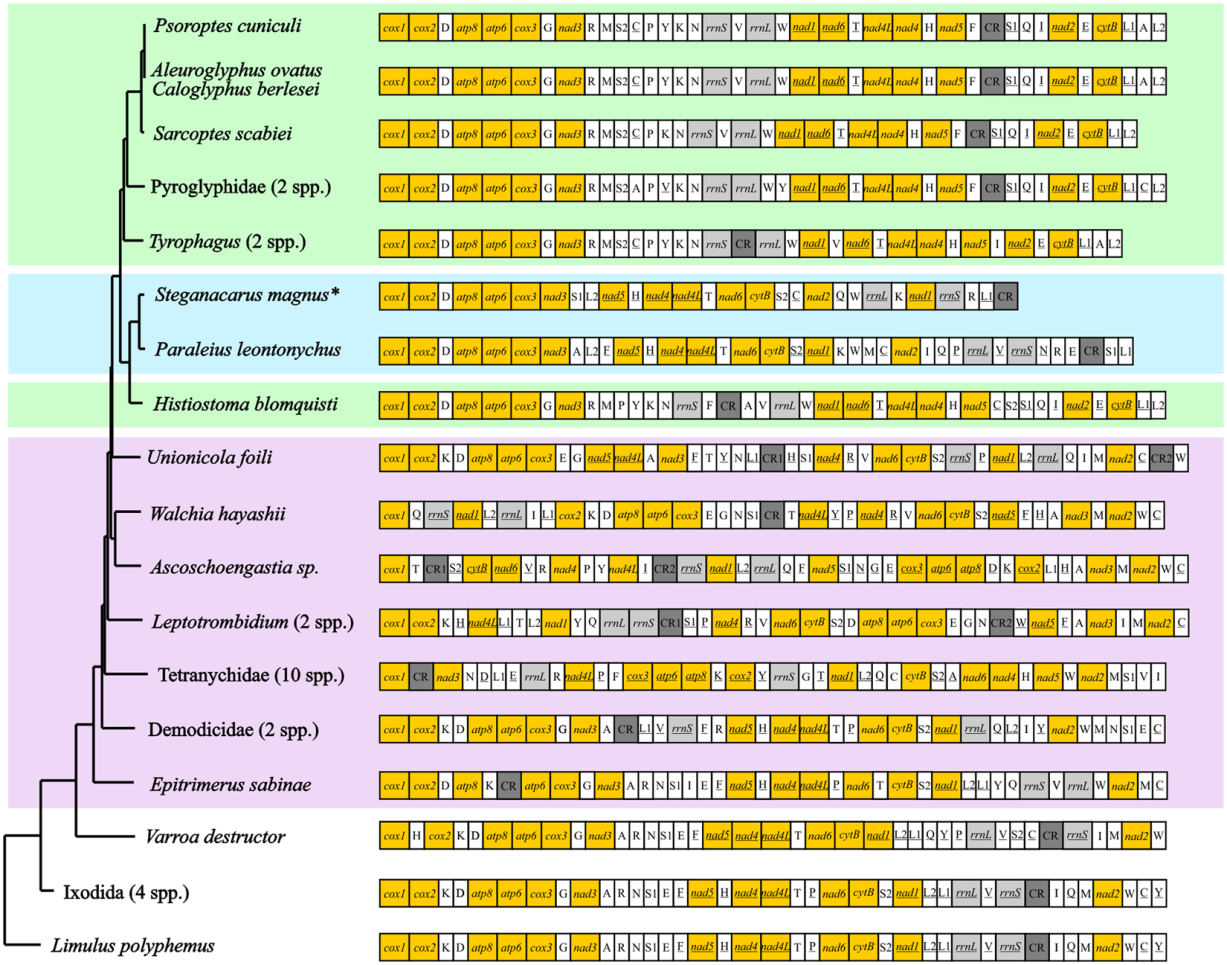
Neighbor joining (NJ) tree based on distances calculated from a CREx gene rearrangement analysis. Genes are drawn in their original order; intergenic distances are not included and sizes of genes are not to scale. Protein-coding genes are colored in yellow, rRNAs in light grey and control regions in dark grey. All abbreviations are the same as in Fig. 1 and Table 1. Genes are transcribed from left to right excepting the underlined ones, which are located on the (−)-strand. *Gene annotation of the present study was used for this analysis.

The Neighbor joining (NJ) tree obtained from gene rearrangement analyses also suggested that the Sarcoptiformes evolved within the Trombidiformes (Fig. 5). The exact branching order, however, differed from the tree topologies obtained from ND and AAD dataset. Interestingly, the two oribatid species *P. leontonychus* and *S. magnus* clustered together despite their obvious differences in the number of tRNA genes present. *Histiosoma blomquisti* was placed as sister taxon of the two oribatid species, rendering the Astigmata paraphyletic based on gene rearrangement patterns.

## Discussion

### General aspects of the new acariform mitogenome

The mitogenome of *P. leontonychus* is the second published complete oribatid mite mitogenome so far and differs clearly from the previously described one from *S. magnus*, not only because of differences in the gene arrangement (Fig. 3) but also in the number of identified tRNAs (for details see below). As in other acariform mites, extensive gene order rearrangement became evident in *P. leontonychus* (Figs 3 and 5). However, compared with other sarcoptiform mites, it has the second least rearranged mitogenome compared to *L. polyphemus*, the hypothetical ancestor, according to the number of breakpoints calculated via CREx analysis (Supplementary Table S3). The least rearranged genome appears to be that of the second oribatid species, *S. magnus* (Fig. 5). Concerning PCGs, *P. leontonychus* has the same gene arrangement as *S. magnus*, with the exception of *nad1* and *nad2,* which changed position and strand (Figs 3 and 5). All other species are not closely related to our study species and show multiple rearrangements of gene order and placement. However, there is one consistent gene arrangement within all studied Sarcoptiformes, namely *cox1-cox2- tRNA*^*Asp*^*-atp8-atp6-cox3*-[*tRNA*^*Gly*^(only in Astigmata)]-*nad3* (Fig. 5), indicating a potential ancestral pattern within this order. Also interesting is the comparison of used start/stop codons between the two Oribatida: For *atp6* and *nad5*, both species use the same start and stop codons; otherwise there are only four further PCGs for which either the same start or stop codon is used in *P. leontonychus* and *S. magnus* (*nad3* the start codon ATT; *cox3*, *nad4L* and *nad6* the stop codon TAA).

In Acariformes, the nucleotide composition of the (+)-strand is generally biased towards A and T, with an average A + T-content of about 75%^35^. This is also true for *P. leontonychus* where there is a clear excess of A + T against G + C nucleotides. However, compared to the other described mite mitogenomes, our study species has - with 64.4% - the lowest A + T content^35,46^. In general, metazoan species show a clear strand asymmetry in nucleotide composition: the leading strand is biased in favor of A and C and consequently, the lagging strand in favor of T and G^47^. In *P. leontonychus*, the genome has a positive AT-skew and a negative GC-skew of the leading strand, which is similar to other arthropods^48–50^. This is particularly true for Acari, with the mitogenomes of both Acariformes and Parasitiformes usually having negative GC-skews^35^^,^^46,51^. As there is no general trend in strand composition in acariform mites, it is not surprising that it differs also between *S. magnus* and *P. leontonychus*. There, both AT- and GC-skews are negative on the leading strand^29^, indicating a reverse strand-compositional bias of the genome, i.e., meaning an excess of (i) T relative to A and (ii) G relative to C nucleotides.

### New insights into tRNA evolution and the pitfalls of their annotation

tRNAs are characterized by their conserved secondary structure with the characteristic cloverleaf layout with a 7 bp acceptor stem, a 5 bp anticodon stem and a D- and a T-arm. This conserved structure is the basis of several available prediction tools including tRNAscan-SE^43^, ARWEN^44^ and MiTFi^22^, where the latter two were specifically developed for predicting tRNAs in mitochondrial genomes. Prediction is very reliable for tRNAs corresponding to the cloverleaf structure. However, identification of tRNAs lacking one or both arms or containing mismatches in the stems is challenging. Either such tRNAs are missed during prediction or predicted with an implausible secondary structure. In addition, multiple tRNAs with different anticodons at almost the same genomic position are predicted (either on the same or opposite strand). In the current study, we could identify 18 tRNAs using prediction tools and further 2 by manual annotation. Predictions of the different tools were in some cases contradictory, which had to be resolved by calculating the unconstrained and constrained MFEs as a proxy for the stability and selecting the sequence and structure with the smaller MFE. For *S. magnus* - the only Oribatida mitogenome available up to now – only 6 tRNAs have been annotated in the initial publication^29^. Klimov and OConnor^27^ could identify another two tRNAs (*tRNA*^*Ala*^, and *tRNA*^*Lys*^) and re-annotate three of the previously described ones (*tRNA*^*Pro*^->*tRNA*^*Thr*^, *tRNA*^*Trp*^->*tRNA*^*Ser2*^, *tRNA*^*Ser2*^->*tRNA*^*Trp*^) based on MFE values (Fig. 3). In our study, we could confirm the prediction of these tRNAs and could add four additional ones, mainly based on predictions by MITOS2^52^.

Nevertheless, our data suggest that besides three other sarcoptiform species, the two available oribatid mt genomes have a reduced set of tRNAs. Both oribatid species have lost tRNA^*Tyr*^ as well as *tRNA*^*Gly*^ and in addition, *S. magnus* lacks eight further tRNAs (*tRNA*^*Ala*^, *tRNA*^*Asn*^, tRNA^Glu^, *tRNA*^*Ile*^, *tRNA*^*Met*^, *tRNA*^*Phe*^, *tRNA*^*Pro*^, *tRNA*^*Val*^). However, the loss of tRNA genes follows no specific rule and can be quite variable within groups, even between closely related species^18,20^, indicating that this loss occurred independently many times in the tree of life.

During acariform evolution, tRNAs appear to have gradually lost either the D- or T-arm or both, leaving only *tRNA*^*Lys*^ with the typical cloverleaf structure in all currently known mitogenomes (Fig. 4). This is especially true for the Astigmata, which retain only *tRNA*^*Lys*^ with the typical cloverleaf structure. *Paraleius leontonychus*, as a member of the Oribatida, has five tRNAs with both arms present. Whereas all other sarcoptiform mites analyzed so far have the lowest number of cloverleaf-like mt-tRNAs among mites, the number of tRNAs with two arms is much higher in *P. leontonychus* and in the range typical for trombidiform mites. Hence, the apparent increased reduction of tRNA arms in sarcoptiform mites appears to be at least in part due to a taxon sampling bias. In this context, we got another unexpected result concerning the secondary structure of the tRNA for cysteine. While *tRNA*^*Cys*^, like *tRNA*^*Phe*^ and *tRNA*^*His*^, lacks the T-arm in all known Acariformes species^28^, it has the typical cloverleaf structure in both oribatids, *P. leontonychus* and *S. magnus* (here it is the only intact tRNA). Whether this is an ancestral feature in Oribatida, in general, remains questionable as both taxa do not represent basal species of this suborder^45^. However, our result indicates that acariform mites might have lost the T-arm in *tRNA*^*Cys*^ multiple times independently, contradicting the hypothesis of Xue *et al*.^28^ that the T-arm loss in *tRNA*^*Cys*^ is likely ancestral in acariform mites. The lack of the T-arm in the *tRNA*^*Phe*^ and *tRNA*^*His*^ of *P. leontonychus* is congruent with the pattern in other Acariformes, which supports Xue *et al*.’s^28^ hypothesis that truncation of these two tRNAs occurred once in the most recent common ancestor of Acariformes.

Besides the atypical secondary structure of tRNAs, there are further interesting phenomena which complicate a straight forward tRNA annotation. One well-known characteristic throughout metazoans^22,53^ is that many mt-tRNA genes overlap with other genes. This is particularly true for arthropods^22,33,54^, and thus, not surprising, also the case in *P. leontonychus*. In our study species, we further found mismatched base pairs and/or truncated acceptor stems. Truncated acceptor stems (with fewer than seven paired bases) but also general stem mismatches have been already reported in arthropods and velvet worms^22,23,35,54,55^. Within the Acari, examples for such aberrant acceptor stems can be found in acariform mites, as in the genera *Dermatophagoides*, *Leptotrombidium* and *Panonychus*^27,28,56^, or in the spider mite genus *Tetranychus*^51^. To allow these tRNAs to function, a posttranscriptional RNA editing process, which restores the truncated acceptor stem in mt-tRNAs, has been previously shown to exist^57^. Additionally, Yokobori and Pääbo^58,59^ showed that RNA editing further occurs when there is an overlap of tRNA acceptor stem and PCG encoded on the same strand. Both cases, mismatches in the acceptor stem and overlap with a PCG were also found in three species of the *Habronattus* spider^60,61^ and it was postulated that a similar RNA editing mechanism could exist there too. Whether similar processes play a role in *P. leontonychus*, remains to be seen. In nematodes it was previously shown that tRNAs with an extreme truncated structure are still functional because of a gene duplication of the elongation factor EF-Tu. For example, in *Caenorhabditis elegans* nuclear DNA encodes two elongation factor EF-Tu homologs, EF-Tu1 and EF-Tu2, whereof EF-Tu1 binds to T-arm-lacking and EF-Tu2 to D-arm-lacking tRNAs only^62,63^.

### Phylogenetic relationship of Sarcoptiformes

Inferring “true” phylogenetic affinities and classification within the Acariformes has been a longstanding challenge. For example, the paraphyly of Trombidiformes (also supported by our phylogeny) and its consequences has been recently discussed in the course of mitogenomic studies^28,64^. Consistent with several previous studies, our phylogenetic reconstruction based on the 13 mt-PCGs inferred the origin of Astigmata within Oribatida. In general, the origin of the Astigmata is a particularly widely discussed topic and several authors tried to answer this question by employing a variety of approaches^28,65–67^. General historical concepts of relationships between Trombidiformes, Oribatida and Astigmata have been summarized by Norton^68^. Among the various different concepts put forward in the past, there are two widely established hypotheses: the first considers that both Oribatida and Astigmata are monophyletic sister groups^69,70^, and the second assumes that a lineage within Oribatida is the sister group of Astigmata^37,68,71^. In the classification by Lindquist *et al*.^37^, the acariform order Sarcoptiformes is divided into the two suborders Endeostigmata and Oribatida, whereof the latter one comprises five supercohorts: the most primitive Palaeosomatides, the early-derived Enarthronotides and Parhyposomatides and the middle-to-highly derived Mixonomatides and Desmonomatides. Our results are congruent with the findings of Dabert *et al*.^65^, who investigated the molecular phylogeny of acariform mites using sequences of the nuclear small subunit rRNA gene (18S rDNA) and COI amino acid data and found that Astigmata evolved within the Desmonomatides. Other molecular genetic studies either suggested a within-Oribatida origin for Astigmata^72^ or rejected it^45^. Moreover, investigations based on different morphological traits^68^ as well as on the chemical composition of opisthonotal gland secretions^73^ provided an indication of an astigmatan evolution within the Oribatida. A recent study, however, inferred based on sequences of the small and large subunits of nuclear rDNA that Astigmata and “traditional” Desmonomatides are most likely reciprocally monophyletic sister groups^67^. The logical next steps will be the integration of more sarcoptiform mitogenomes including species from each of the five supercohorts. We suppose that especially species of the basal desmonomatan Nothrina, plus basal Brachypylina (e.g. Hermannielloidea, Neoliodoidea) might be helpful to get a clearer picture of the within-Oribatida evolution of Astigmata. In addition, nuclear multilocus, and in particular genome scale data, would even further increase the resolution of ambiguous relationships and provide a robust phylogenetic framework of acariform mite relationships for comparative phylogenetic analyses in the hopefully near future.

## Conclusions

The newly sequenced mitogenome of the oribatid mite *P. leontonychus* has important ramifications for our understanding of mitogenome evolution in sarcoptiform mites. It appears that throughout the acariform tree tRNAs have gradually lost either D- or T-arm or both. The previously reported extreme paucity of complete cloverleaf-like tRNAs in sarcoptiform as compared to trombidiform mites might be, at least in part, due to a taxon sampling bias as the number of cloverleaf-like tRNAs in the newly sequenced *P. leontonychus* falls well within the range typical for the Trombidiformes. Phylogenetic mitogenomic analyses suggest paraphyly of the Oribatida with respect to the Astigmata. However, as the mitochondrial genome is essentially just one single locus, potentially impacted by (ancient) incomplete lineage sorting^74^, nuclear multilocus data will be necessary, together with an increased taxon sampling, to confirm these relationships within the Sarcoptiformes and provide a robust phylogenetic framework for the acariform mites.

## Methods

### Sampling and DNA-extraction

*Paraleius leontonychus* was collected from a bark sample of *Picea abies* infested by different bark beetle species in Paldau (Styria, Austria; 46°55′53.0″N 15°45′54.0″E), in autumn 2015. Specimens were extracted alive with a Berlese-Tullgren funnel and preserved in 100% ethanol for further investigation.

Whole genomic DNA was extracted from a single mite individual using the QIAamp DNA Mini Kit (Qiagen, Hilden, Germany) following the manufacture’s protocol. Purified DNA was eluted in a single step in 50 µl HPLC water. After DNA extraction, the sclerotized body remnants were mounted on permanent slides as voucher.

### Library preparation and sequencing

Total genomic DNA was quantified using the QuantiFluor® dsDNA Dye on a Quantus™ Fluorometer (Promega, Mannheim, Germany). For library preparation with the NEBNext® Ultra II DNA Library Prep Kit for Illumina® (New England BioLabs, Frankfurt, Germany) with the NEBNext® Multiplex Oligos for Illumina® (Index Primers Set 1) according to manufacturer’s instructions 1 ng total DNA was randomly fragmented by ultrasonication in a microTUBE on a M220 Focused-ultrasonicator™ (Covaris, Woburn, MA, USA) according to Thannesberger *et al*.^75^. End repair and adapter ligation were performed according to manufacturer’s instructions and size selection and PCR amplification with 12 cycles according to Thannesberger *et al*.^75^. The library was purified and eluted in 30 µl 1 x TE pH 8.0 and the quality was examined on an Agilent BioAnalyzer High Sensitivity DNA chip (Agilent Technologies, CA, USA) and again quantified on a Quantus™ Fluorometer (Promega, Mannheim, Germany). The final library was sequenced at 8 pM with 5% PhiX with v3 600 cycles chemistry on an Illumina MiSeq desktop sequencer in paired end mode. FastQ raw data were used for sequence analysis.

### De novo assembly and annotation

Raw sequences were quality controlled with fastQC^76^. Filtering and assembly was performed in CLC Genomics Workbench (version 6.5.2, CLC bio, Aarhus, Denmark). The contig representing the mitogenome was identified with a BLAST^77^ search of the *S. magnus* mitogenome against all contigs in the assembly. A missing sequence stretch between 16S and 12S rRNAs (13739 .. 13894) was identified by mapping the raw reads against the draft mt genome. This gap was closed with Sanger sequences of the amplicon generated with three primer pairs (Supplementary Table S4). The assembled genome was annotated using the MITOS WebServer under the mitochondrial genetic code for invertebrates (revision 656; http://mitos.bioinf.uni-leipzig.de^6^). The resulting annotation was curated manually. As the MITOS prediction for the 16S rRNA comprised only 534 bps, we extracted the 16S sequences from the available Sarcoptiformes mitogenomes (Supplementary Table S5) and performed a multiple sequence alignment with MUSCLE^78^ and extended the 16S gene based on the observed conserved regions. Secondary structures in the control region were identified using the Mfold web server (http://unafold.rna.albany.edu/?q=mfold/DNA-Folding-Form)^79^.

To extend the tRNA-predictions provided by MITOS with the MiTFi approach^22^, we also applied the MITOS2 webserver (revision 941; http://mitos2.bioinf.uni-leipzig.de^52^), tRNAscan-SE^43^ (version 1.3.1) and ARWEN^44^ (version 1.2.3) on the mitogenome sequence. Parameters for MITOS2 were: Reference: ‘MetazoaRefSeq. 63’, Genetic Code: ‘5 Invertebrate’, Feature types: ‘PCG,’ ‘tRNA’, rRNA’. tRANscan-SE was invoked with the following parameters:-Q -O -H -D -X 0.1 -g gcode.invmito. ARWEN parameters were: -c -gcinvert -w -seq –br. tRNAs predicted with a non-standard mitochondrial anticodon were removed (with the exception of S1 with GCT). To select the most probable of alternative structure predictions, the constrained and unconstrained minimum free energy (MFE) was calculated using RNAeval and RNAfold from the Vienna RNA Package (version 2.3.3) respectively^80^. The secondary structure with the smallest constrained MFE was considered the most likely one.

To define the extent of gene rearrangement in acariform mitogenomes from the ancestral arthropod ground pattern of *L. polyphemus*, breakpoints were calculated using the web-based program CREx^81^.

A map of the mitochondrial genome was drawn with the CGView Server (http://stothard.afns.ualberta.ca/cgview_server ^82^).

Strand bias in nucleotide composition was calculated as GC- and AT-skews, as (G − C)/(G + C) and (A – T)/(A + T), respectively^83^.

### Phylogenetic and gene rearrangement analyses

To infer the phylogenetic position of *P. leontonychus* within the Acariformes, we generated a data set of 37 mite taxa (six Parasitiformes and 31 Acariformes, Supplementary Table S5) which included only those species with a complete set of 13 PCGs. First, sequences of each PCG were aligned separately using the TranslatorX server (http://translatorx.co.uk ^84^), where MAFFT^85^ is used to build the protein alignment. Translation was done under the invertebrate mt genetic code. Additionally, poorly aligned sites were excluded using the alignment cleaning program GBlocks (implemented within TranslatorX) under the default parameters^86^. Finally, the single alignments were concatenated into one data set, with a final length of 6,879 bp, using DAMBE 5.5.24^87^.

The datasets generated and/or analyzed in the current study are available from the corresponding author on request.

All analyses were performed using data sets of both nucleotide (ND) and amino acid (AAD) sequences of the PCGs, which were partitioned by genes and by codon positions. To select the best-fitting partitioning scheme and models of evolution, we used PartitionFinder v2^88,89^ with the settings: (i) unlink branch lengths and (ii) use the corrected Akaike information criterion (AICc) for model selection. ML and BI analyses were performed using the RAxML web-server (http://embnet.vital-it.ch/raxml-bb/index.php ^90^) and MrBayes v3.2.4^91^ under the best substitution models and partition scheme identified (Supplementary Table S6). Bayesian analyses were run with the settings nst = 6 and rates = invgamma for the ND and aamodelpr = fixed(wag) for the AAD. Posterior probabilities were obtained from a Metropolis-coupled Markov chain Monte Carlo simulation conducting two runs simultaneously, each with four chains (one cold, three heated) for 5,000,000 (ND) or 2,000,000 (AAD) generations. Trees were sampled every 1,000 generations and the first 10% were discarded as burn-in. Mixing and convergence of the parameters to stationary distributions were evaluated in Tracer v.1.6^92^. All estimated parameters showed ESS values above 200.

To explore the potential of mitogenomic rearrangements for answering phylogenetic questions, we applied a pairwise comparison approach of the mt gene order of the same mite species as in the phylogenetic analyses. Only the Chilean predatory mite *Phytoseiulus persimilis* was excluded from this analyses because of its extremely reshuffled gene order (35 genes changed position) compared with *L. polyphemus*^25^. The analysis was performed using CREx with default parameters. For a hierarchical grouping of the taxa, the distances were imported into PAST3^93^ and analyzed using the NJ clustering method with Euclidean distance as the similarity index.

## Electronic supplementary material

Supplementary Information
