# Supplementary material for: The mitochondrial genome of the oribatid mite Paraleius leontonychus: new insights into tRNA evolution and phylogenetic relationships in acariform mites
Source: Sci Rep. 2018 May 15;8:7558. doi: 10.1038/s41598-018-25981-w (PMC5954100; doi:10.1038/s41598-018-25981-w)

# The mitochondrial genome of the oribatid mite *Paraleius leontonychus*: new insights into tRNA evolution and phylogenetic relationships in acariform mites

Sylvia Schäffer[1*], Stephan Koblmüller[1], Ingeborg Klymiuk[2], Gerhard G. Thallinger[3,4*]

[1] Institute of Zoology, University of Graz, Universitätsplatz 2, 8010 Graz, Austria

[2] Core Facility Molecular Biology, Center for Medical Research, Medical University of Graz, Graz, Stiftingtalstraße 24, 8010 Graz, Austria

[3] Institute of Computational Biotechnology, Graz University of Technology, Petersgasse 14, 8010 Graz, Austria

[4] OMICS Center Graz, BioTechMed Graz, Stiftingtalstraße 24, 8010 Graz, Austria

* Correspondence to: sylvia.schaeffer@uni-graz.at; gerhard.thallinger@tugraz.at

## Supplementary Tables

**Table S1**. tRNAs of *P. leontonychus* predicted byARWEN, MITOS, MITOS2 and tRNAScan-SE with unconstrained (mfe_u) and constrained (mfe_c) minimum free energy for the predicted sequence and secondary structure. source .. source of prediction; gene .. gene name; product .. transcribed tRNA; start .. start position on sequence, end .. end position on sequence; strand .. strand on sequence; acoff .. offset of anticodon within gene; acstart .. start of anticodon on sequence; slen .. length of gene.

| species | source | gene | product | start | end | strand | anticodon | acoff | acstart | slen | mfe_u | mfe_c |
|---|---|---|---|---|---|---|---|---|---|---|---|---|
| Pl | ARWEN | trn- | tRNA-Thr(ggt) | 2025 | 2094 | -1 | ggt | 38 | 2055 | 70 | -15.1 | -6.5 |
| Pl | M1,M2 | trnD | tRNA-Asp(gtc) | 2208 | 2267 | 1 | gtc | 35 | 2242 | 60 | -5.8 | -2.5 |
| Pl | ARWEN | trn- | tRNA-???(at ) | 2870 | 2925 | -1 | at | 27 | 2897 | 56 | -10.4 | -8 |
| Pl | MANUAL | trnA | tRNA-Ala(tgc) | 4262 | 4306 | 1 | tgc | 20 | 4281 | 45 | -5.4 | -5.4 |
| Pl | ARWEN | trnL2 | tRNA-Leu(taa) | 4307 | 4378 | 1 | taa | 39 | 4345 | 72 | -8.7 | -1.8 |
| Pl | MITOS2 | trnL2 | tRNA-Leu(taa) | 4307 | 4371 | 1 | taa | 39 | 4345 | 65 | -8.7 | -6 |
| Pl | M1,TR | trnL2 | tRNA-Leu(taa) | 4308 | 4370 | 1 | taa | 38 | 4345 | 63 | -7.8 | -7.3 |
| Pl | MITOS1 | trnF | tRNA-Phe(gaa) | 4362 | 4425 | -1 | gaa | 32 | 4392 | 64 | -17.4 | -2.1 |
| Pl | ARWEN | trnF | tRNA-Phe(gaa) | 4370 | 4424 | -1 | gaa | 31 | 4392 | 55 | -9.7 | -3.7 |
| Pl | MITOS2 | trnF | tRNA-Phe(gaa) | 4371 | 4423 | -1 | gaa | 30 | 4392 | 53 | -8.4 | -0.9 |
| Pl | AR,M1,M2 | trnH | tRNA-His(gtg) | 6034 | 6088 | -1 | gtg | 32 | 6055 | 55 | -11 | -2.4 |
| Pl | AR,M1,M2 | trnT | tRNA-Thr(tgt) | 7656 | 7710 | 1 | tgt | 31 | 7686 | 55 | -7 | -3.6 |
| Pl | AR,M1,M2 | trnS2 | tRNA-Ser(tga) | 9234 | 9287 | 1 | tga | 21 | 9254 | 54 | -10.7 | -9.3 |
| Pl | M1,TR | trnK | tRNA-Lys(ttt) | 10184 | 10247 | 1 | ttt | 32 | 10215 | 64 | -12.4 | -11.4 |
| Pl | ARWEN | trnK | tRNA-Lys(ttt) | 10186 | 10246 | 1 | ttt | 30 | 10215 | 61 | -7.2 | -3.9 |
| Pl | ARWEN | trnW | tRNA-Trp(tca) | 10250 | 10313 | 1 | tca | 33 | 10282 | 64 | -5.1 | -0.4 |
| Pl | M1,M2,TR | trnW | tRNA-Trp(tca) | 10250 | 10311 | 1 | tca | 33 | 10282 | 62 | -5.1 | -0.4 |
| Pl | ARWEN | trn- | tRNA-Asn(att) | 10318 | 10389 | -1 | att | 33 | 10355 | 72 | -13 | -0.7 |
| Pl | M1,M2 | trnM | tRNA-Met(cat) | 10340 | 10397 | 1 | cat | 33 | 10372 | 58 | -11.3 | -5.7 |
| Pl | MITOS2 | trnF | tRNA-X__(-) | 10375 | 10451 | -1 | - | | | 77 | -13.2 | -1.3 |
| Pl | ARWEN | trnC | tRNA-Cys(gca) | 10393 | 10463 | -1 | gca | 37 | 10425 | 71 | -13.1 | -4.4 |
| Pl | MITOS1 | trnC | tRNA-Cys(gca) | 10404 | 10451 | -1 | gca | 25 | 10425 | 48 | -5.3 | 4 |
| Pl | MITOS2 | trnC | tRNA-Cys(gca) | 10404 | 10451 | -1 | gca | 25 | 10425 | 48 | -5.3 | -0.8 |
| Pl | ARWEN | trn- | tRNA-Asp(atc) | 11411 | 11470 | -1 | atc | 24 | 11445 | 60 | -8 | 0.3 |
| Pl | M1,M2 | trnI | tRNA-Ile(gat) | 11414 | 11470 | 1 | gat | 32 | 11445 | 57 | -7.2 | -5.4 |
| Pl | ARWEN | trnQ | tRNA-Gln(ttg) | 11468 | 11532 | -1 | ttg | 31 | 11500 | 65 | -12.5 | -8.6 |
| Pl | M1,M2 | trnQ | tRNA-Gln(ttg) | 11469 | 11531 | -1 | ttg | 30 | 11500 | 63 | -11.8 | 99987.2 |

| species | source | gene | product | start | end | strand | anticodon | acoff | acstart | slen | mfe_u | mfe_c |
|---|---|---|---|---|---|---|---|---|---|---|---|---|
| Pl | M1,M2 | trnP | tRNA-Pro(tgg) | 11566 | 11629 | -1 | tgg | 39 | 11589 | 64 | -9.6 | 7 |
| Pl | MANUAL | trnV | tRNA-Val(tac) | 12752 | 12798 | -1 | tac | 22 | 12775 | 47 | -8.5 | -8.5 |
| Pl | ARWEN | trn- | tRNA-Ser(gga) | 12771 | 12837 | -1 | gga | 23 | 12813 | 67 | -9.5 | 4.2 |
| Pl | ARWEN | trnN | tRNA-Asn(gtt) | 13474 | 13531 | -1 | gtt | 30 | 13500 | 58 | -9 | 6.4 |
| Pl | M1,M2 | trnN | tRNA-Asn(gtt) | 13476 | 13531 | -1 | gtt | 30 | 13500 | 56 | -9 | -1.1 |
| Pl | MITOS2 | trnR | tRNA-Arg(tcg) | 13518 | 13563 | 1 | tcg | 30 | 13547 | 46 | -5.9 | 5.8 |
| Pl | TRNASC | trn- | tRNA-Ser(cga) | 13522 | 13581 | -1 | cga | 33 | 13547 | 60 | -6.1 | 7.5 |
| Pl | AR,M1,M2,TR | trnE | tRNA-Glu(ttc) | 13572 | 13633 | 1 | ttc | 31 | 13602 | 62 | -7.2 | -1.8 |
| Pl | ARWEN | trnS1 | tRNA-Ser(gct) | 14069 | 14125 | 1 | gct | 21 | 14089 | 57 | -11.8 | -8.1 |
| Pl | M1,M2 | trnS1 | tRNA-Ser(gct) | 14069 | 14125 | 1 | gct | 21 | 14089 | 57 | -11.8 | -11.8 |
| Pl | AR,M2 | trnL1 | tRNA-Leu(tag) | 14129 | 3 | 1 | tag | 36 | 14164 | 61 | -7 | -5 |

**Table S2**. tRNAs of *S. magnus* predicted by ARWEN, MITOS, MITOS2 and tRNAScan-SE with unconstrained (mfe_u) and constrained (mfe_c) minimum free energy for the predicted sequence and secondary structure. source .. source of prediction; gene .. gene name; product .. transcribed tRNA; start .. start position on sequence, end .. end position on sequence; strand .. strand on sequence; acoff .. offset of anticodon within gene; acstart .. start of anticodon on sequence; slen .. length of gene.

| species | source | gene | product | start | end | strand | anticodon | acoff | acstart | slen | mfe_u | mfe_c |
|---|---|---|---|---|---|---|---|---|---|---|---|---|
| Sm | MITOS2 | trnD | tRNA-Asp(gtc) | 2148 | 2204 | 1 | gtc | 36 | 2183 | 57 | -5.8 | 5.2 |
| Sm | KLIMOV | trnD | tRNA-Asp(gtc) | 2154 | 2220 | 1 | gtc | 30 | 2183 | 67 | -8.2 | 7.7 |
| Sm | MITOS2 | trnM | tRNA-X__(tat) | 3644 | 3699 | 1 | tat | 29 | 3672 | 56 | -4.4 | 5.4 |
| Sm | KLIMOV | trnG | tRNA-Gly(tcc) | 3773 | 3841 | 1 | tcc | 31 | 3803 | 69 | -7.8 | 6.1 |
| Sm | MITOS2 | trnP | tRNA-Pro(tgg) | 3934 | 3987 | 1 | tgg | 29 | 3962 | 54 | -4.4 | 12.8 |
| Sm | ARWEN | trnS1 | tRNA-Ser(tct) | 4184 | 4256 | 1 | tct | 34 | 4217 | 73 | -14.5 | 4.2 |
| Sm | MITOS2 | trnS1 | tRNA-Ser(tct) | 4199 | 4248 | 1 | tct | 19 | 4217 | 50 | -9.2 | -5.5 |
| Sm | MITOS2 | trnF | tRNA-Phe(gaa) | 4222 | 4304 | -1 | gaa | 33 | 4270 | 83 | -8.7 | 9.4 |
| Sm | MITOS2 | trnL2 | tRNA-Leu(taa) | 4279 | 4347 | 1 | taa | 30 | 4308 | 69 | -10.7 | -2.3 |
| Sm | AR,DO | trnL2 | tRNA-Leu(taa) | 4289 | 4339 | 1 | taa | 20 | 4308 | 51 | -6.9 | -3.8 |
| Sm | MITOS2 | trnA | tRNA-X__(agc) | 5371 | 5445 | -1 | agc | 16 | 5428 | 75 | -13.3 | 1.5 |
| Sm | MITOS2 | trnH | tRNA-His(gtg) | 5930 | 5992 | -1 | gtg | 27 | 5964 | 63 | -9.7 | 13.1 |
| Sm | DOMES | trnH | tRNA-His(gtg) | 5941 | 5997 | -1 | gtg | 32 | 5964 | 57 | -9.7 | 1 |
| Sm | MITOS2 | trnV | tRNA-X__(aac) | 6240 | 6303 | 1 | aac | 47 | 6286 | 64 | -10.3 | -0.3 |
| Sm | MITOS2 | trnN | tRNA-X__(att) | 6262 | 6323 | -1 | att | 21 | 6301 | 62 | -14 | -4.9 |
| Sm | TRNASC | trnL1 | tRNA-Leu(tag) | 6748 | 6815 | 1 | tag | 30 | 6777 | 68 | -6.4 | 4.9 |
| Sm | MITOS2 | trnT | tRNA-Thr(tgt) | 7503 | 7573 | 1 | tgt | 38 | 7540 | 71 | -11 | 100003.8 |
| Sm | KLIMOV | trnT | tRNA-Thr(tgt) | 7521 | 7562 | 1 | tgt | 20 | 7540 | 42 | -7.5 | -5.6 |
| Sm | DOMES | trnM | tRNA-Met(tat) | 7526 | 7581 | -1 | agg | 33 | 7547 | 56 | -5.7 | 16.2 |
| Sm | TRNASC | trnW | tRNA-Trp(tca) | 9037 | 9113 | -1 | tca | 35 | 9077 | 77 | -8.7 | 2.9 |
| Sm | KLIMOV | trnS2 | tRNA-Ser(tga) | 9039 | 9113 | 1 | tga | 39 | 9077 | 75 | -10.6 | 3.6 |
| Sm | DOMES | trnW | tRNA-Trp(tca) | 9051 | 9106 | -1 | tca | 28 | 9077 | 56 | -6.7 | 5.6 |
| Sm | KLIMOV | trnS2 | tRNA-Ser(tga) | 9056 | 9110 | 1 | tga | 22 | 9077 | 55 | -8.5 | 3.9 |
| Sm | ARWEN | trnC | tRNA-Cys(gca) | 9071 | 9142 | -1 | gca | 27 | 9114 | 72 | -9.3 | 1.7 |
| Sm | MITOS2 | trnC | tRNA-Cys(gca) | 9071 | 9143 | -1 | gca | 28 | 9114 | 73 | -9.3 | 4.3 |
| Sm | ARWEN | trnF | tRNA-Phe(gaa) | 9926 | 10004 | -1 | gaa | 37 | 9966 | 79 | -14.7 | -4.1 |
| Sm | ARWEN | trnQ | tRNA-Gln(ttg) | 10133 | 10197 | -1 | ttg | 37 | 10159 | 65 | -11.4 | 5.4 |

| species | source | gene | product | start | end | strand | anticodon | acoff | acstart | slen | mfe_u | mfe_c |
|---|---|---|---|---|---|---|---|---|---|---|---|---|
| Sm | MITOS2 | trnQ | tRNA-Gln(ttg) | 10134 | 10196 | -1 | ttg | 36 | 10159 | 63 | -10.8 | 5.6 |
| Sm | DOMES | trnQ | tRNA-Gln(ttg) | 10136 | 10202 | -1 | ttg | 42 | 10159 | 67 | -10.1 | -2.2 |
| Sm | MITOS2 | trnY | tRNA-Tyr(gta) | 10157 | 10219 | -1 | gta | 23 | 10195 | 63 | -8.6 | 7.4 |
| Sm | ARWEN | trnS2 | tRNA-Ser(tga) | 10248 | 10304 | -1 | tga | 25 | 10278 | 57 | -19 | -7 |
| Sm | DOMES | trnS2 | tRNA-Ser(tga) | 10248 | 10304 | -1 | tga | 25 | 10278 | 57 | -19 | -5.5 |
| Sm | MITOS2 | trnS2 | tRNA-Ser(tga) | 10252 | 10300 | -1 | tga | 21 | 10278 | 49 | -13.2 | -6.4 |
| Sm | MITOS2 | trnW | tRNA-Trp(tca) | 10253 | 10301 | 1 | tca | 26 | 10278 | 49 | -9.6 | -8.1 |
| Sm | MITOS2 | trnE | tRNA-Glu(ttc) | 10613 | 10683 | -1 | ttc | 39 | 10643 | 71 | -8.3 | 10.5 |
| Sm | ARWEN | trnL2 | tRNA-Leu(taa) | 10774 | 10833 | 1 | taa | 29 | 10802 | 60 | -15.6 | 1.9 |
| Sm | MITOS2 | trnG | tRNA-Gly(tcc) | 10825 | 10881 | -1 | tcc | 24 | 10856 | 57 | -5 | 200001.3 |
| Sm | MITOS2 | trnK | tRNA-Lys(ttt) | 10932 | 10986 | -1 | ttt | 38 | 10947 | 55 | -4.8 | 2.7 |
| Sm | ARWEN | trnQ | tRNA-Gln(ttg) | 10980 | 11037 | -1 | ttg | 21 | 11015 | 58 | -3.8 | 3.6 |
| Sm | KLIMOV | trnK | tRNA-Lys(ttt) | 11238 | 11301 | 1 | ttt | 36 | 11273 | 64 | -9 | 9.7 |
| Sm | MITOS2 | trnI | tRNA-X__(aat) | 12444 | 12535 | 1 | aat | 17 | 12460 | 92 | -15.6 | 5.7 |
| Sm | MITOS2 | trnR | tRNA-Arg(tcg) | 12780 | 12837 | 1 | tcg | 30 | 12809 | 58 | -1.6 | 8.6 |
| Sm | MITOS2 | trnL1 | tRNA-Leu(tag) | 12880 | 12928 | -1 | tag | 23 | 12904 | 49 | -12.2 | -1.8 |
| Sm | ARWEN | trnF | tRNA-Phe(gaa) | 12902 | 12969 | -1 | gaa | 29 | 12939 | 68 | -6.1 | 3.7 |
| Sm | ARWEN | trnF | tRNA-Phe(gaa) | 13775 | 27 | 1 | gaa | 35 | 13809 | 71 | -8.9 | 0.6 |

**Table S3.** Calculated breakpoints of a CREx analysis to define the extent of mitochondrial gene rearrangement of 18 mites from their hypothetical ancestor *Limulus polyphemus*. Acariform taxa are highlighted in red. * marks species of the order Sarcoptiformes.

| | *Limulus polyphemus* |
|---|---|
| *Aleuroglyphus ovatus=Caloglyphus berlesei** | 25 |
| *Ascoschoengastia sp.* | 27 |
| Demodicidae (2 spp.) | 20 |
| Pyroglyphidae (2 spp.)* | 27 |
| *Epitrimerus sabinae* | 20 |
| *Histiostoma blomquisti** | 27 |
| *Leptotrombidium* (2 spp.) | 29 |
| Ixodida (4 spp.) | 4 |
| *Paraleius leontonychus** | 21 |
| *Psoroptes cuniculi** | 25 |
| *Sarcoptes scabiei** | 22 |
| *Steganacarus magnus** | 17 |
| Tetranychidae (10 spp.) | 32 |
| *Tyrophagus* (2 spp.)* | 25 |
| *Unionicola foili* | 28 |
| *Varroa destructor* | 18 |
| *Walchia hayashii* | 28 |

**Table S4.** Primers used to amplify a missing sequence stretch between positions 13739..13894.

| Segment | fwd | Sequence 5´to 3´ | rev | Sequence 5´to 3´ | Reference | Ta |
|---|---|---|---|---|---|---|
| **16S** | Pl_16Sfwd1 | ctgggacctcaaacgaattaaaatcac | Pl_16Srev1 | gagttcragccggattaaccc | this study | 53°C |
| **16S-12S** | Pl_16Sfwd2 | ccgtgctcttacaccgatttgc | Pl_16Srev2 | cgctaccttagcacagtctaaaccat | this study | 53°C |
| **12S** | Pl_12Sfwd | cggggtctctttgtctacaatcc | Pl_12Srev | ccgtcattcttttgatagacaagtcg | this study | 63°C |
| **12S** | Pl_12Sint | cccgaatttctactcagtcacaaat | | | this study | * |

fwd, forward primer; rev, reverse primer; *, primer used only for sequencing.

**Table S5**. Mite species used in the present study.

| Superorder | Order | Family | Species | GenBank No. | Reference |
|---|---|---|---|---|---|
| Parasitiformes | Ixodida | Argasidae | *Argas africolumbae* | NC_019642.1 | unpublished |
| | | | *Otobius megnini* | NC_023370.1 | [1] |
| | | Ixodidae | *Ixodes uriae* | NC_006078.1 | [2] |
| | | Nuttalliellidae | *Nuttalliella numaqua* | NC_019663.1 | [3] |
| | Mesostigmata | Phytoseiidae | *Phytoseiulus persimilis* | NC_014049.1 | [4] |
| | | Varroidae | *Varroa destructor* | NC_004454.2 | [5] |
| Acariformes | Trombidiformes | Demodicdiae | *Demodex brevis* | NC_026101.1 | [6] |
| | | | *Demodex folliculorum* | NC_026102.1 | [6] |
| | | Eriophyidae | *Epitrimerus sabinae* | NC_029208.1 | [7] |
| | | | *Phyllocoptes taishanensis* | NC_029209.1 | [7] |
| | | Tetranychidae | *Panonychus citri* | NC_014347.1 | [8] |
| | | | *Panonychus ulmi* | NC_012571.1 | unpublished |
| | | | *Tetranychus cinnabarinus* | NC_014399.1 | unpublished |
| | | | *Tetranychus kanzawai* | NC_024676.1 | [9] |
| | | | *Tetranychus ludeni* | NC_024677.1 | [9] |
| | | | *Tetranychus malaysiensis* | NC_024678.1 | [9] |
| | | | *Tetranychus phaselus* | NC_024679.1 | [9] |
| | | | *Tetranychus pueraricola* | NC_024680.1 | [9] |
| | | | *Tetranychus truncatus* | NC_024874.1 | [9] |
| | | | *Tetranychus urticae* | NC_010526.1 | [8] |
| | | Trombiculidae | *Ascoschoengastia sp. TATW-1* | NC_010596.1 | unpublished |
| | | | *Leptotrombidium akamushi* | NC_007601.1 | [10] |
| | | | *Leptotrombidium deliense* | NC_007600.1 | [10] |
| | | | *Walchia hayashii* | NC_010595.1 | unpublished |
| | | Unionicolidae | *Unionicola foili* | NC_011036.1 | [11] |
| | | | *Unionicola parkeri* | NC_014683.1 | [12] |
| | Sarcoptiformes | Acaridae | *Aleuroglyphus ovatus* | NC_023778.1 | [13] |

| | | | | |
|---|---|---|---|---|
| | | *Caloglyphus berlesei* | NC_024637.1 | [14] |
| | | *Tyrophagus longior* | NC_028725.1 | [15] |
| | | *Tyrophagus putrescentiae* | NC_026079.1 | [16] |
| | Histiostomatidae | *Histiostoma blomquisti* | NC_031377.1 | [17] |
| | Pyroglyphidae | *Dermatophagoides farinae* | NC_013184.1 | [18] |
| | | *Dermatophagoides pteronyssinus* | NC_012218.1 | [19] |
| | Sarcoptidae | *Sarcoptes scabiei* | JXLN01000001.1 | [20] |
| | Scheloribatidae | *Paraleius leontonychus* | | *this study* |
| | Steganacaridae | *Steganacarus magnus* | NC_011574.1 | [21] |
| | Psoroptidae | *Psoroptes cuniculi* | NC_024675.1 | [22] |

References


1. Burger, T. D., Shao, R., Labruna, M. B. & Barker, S. C. Molecular phylogeny of soft ticks (Ixodida: Argasidae) inferred from mitochondrial genome and nuclear rRNA sequences. *Ticks and tick-borne diseases* **5**, 195-207 (2014).
2. Shao, R. F., Mitani, H., Barker, S. C., Takahashi, M. & Fukunaga, M. Novel mitochondrial gene content and gene arrangement indicate illegitimate inter-mtDNA recombination in the chigger mite, *Leptotrombidium pallidum*. *J. Mol. Evol.* **60** (2005).
3. Mans, B. J., de Klerk, D., Pienaar, R., de Castro, M. H. & Latif, A. A. The mitochondrial genomes of *Nuttalliella namaqua* (Ixodoidea: Nuttalliellidae) and *Argas africolumbae* (Ixodoidae: Argasidae): estimation of divergence dates for the major tick lineages and reconstruction of ancestral blood-feeding characters. *PLoS One* **7**, e49461 (2012).
4. Dermauw, W., Vanholme, B., Tirry, L. & Van Leeuwen, T. Mitochondrial genome analysis of the predatory mite *Phytoseiulus persimilis* and a revisit of the Metaseiulus occidentalis mitochondrial genome. Genome 53, 285-301 (2010).
5. Navajas, M., Conte, Y. L., Solignac, M., Cros-Arteil, S. & Cornuet, J. The complete sequence of the mitochondrial genome of the honeybee ectoparasite mite *Varroa destructor* (Acari: Mesostigmata). *Mol. Biol. Evol.* **19**, 2313-2317 (2002).
6. Palopoli, M. F., Minot, S., Pei, D., Satterly, A. & Endrizzi, J. Complete mitochondrial genomes of the human follicle mites *Demodex brevis* and *D. folliculorum*: novel gene arrangement, truncated tRNA genes, and ancient divergence between species. *BMC Genomics* **15**, 1124 (2014).

7. Xue, X. F., Guo, J. F., Dong, Y., Hong, X. Y. & Shao, R. Mitochondrial genome evolution and tRNA truncation in Acariformes mites: new evidence from eriophyoid mites. *Sci. Rep.* **6**, 18920 (2016).
8. Van Leeuwen, T. *et al*. Parallel evolution of cytochrome b mediated bifenazate resistance in the citrus red mite *Panonychus citri*. *Insect Mol. Biol.* **20**, 135-140 (2011).
9. Chen, D. *et al*. The complete mitochondrial genomes of six species of *Tetranychus* provide insights into the phylogeny and evolution of spider mites. *PloS one* **9**, e110625 (2014).
10. Shao, R., Barker, S. C., Mitani, H., Takahashi, M. & Fukunaga, M. Molecular mechanisms for the variation of mitochondrial gene content and gene arrangement among chigger mites of the genus *Leptotrombidium* (Acari: Acariformes). *J. Mol. Evol.* **63** (2006).
11. Ernsting, B. R., Edwards, D. D., Aldred, K. J., Fites, J. S. & Neff, C. R. Mitochondrial genome sequence of *Unionicola foili* (Acari: Unionicolidae): a unique gene order with implications for phylogenetic inference. *Experimental and Applied Acarology* **49**, 305 (2009).
12. Edwards, D. D., Jackson, L. E., Johnson, A. J. & Ernsting, B. R. Mitochondrial genome sequence of *Unionicola parkeri* (Acari: Trombidiformes: Unionicolidae): molecular synapomorphies between closely-related *Unionicola* gill mites. *Experimental and applied acarology* **54**, 105-117 (2011).
13. Sun, E., Li, C., Nie, L. & Jiang, Y. The complete mitochondrial genome of the brown leg mite, *Aleuroglyphus ovatus* (Acari: Sarcoptiformes): evaluation of largest non-coding region and unique tRNAs. *Experimental and Applied Acarology* **64**, 141-157 (2014).
14. Sun, E., Li, C., Li, S., Gu, S. & Nie, L. Complete mitochondrial genome of *Caloglyphus berlesei* (Acaridae: Astigmata): The first representative of the genus *Caloglyphus*. *J. Stored Prod. Res.* **59**, 282-284 (2014).
15. Yang, B. & Li, C. Characterization of the complete mitochondrial genome of the storage mite pest *Tyrophagus longior* (Gervais) (Acari: Acaridae) and comparative mitogenomic analysis of four acarid mites. *Gene* **576**, 807-819 (2016).
16. Que, S. *et al*. Complete mitochondrial genome of *Aleuroglyphus ovatus (*Acari: Acaridae). *Mitochondrial DNA Part A* **27**, 563-564 (2016).
17. Lee, C. & Wang, J. The complete mitochondrial genome of *Histiostoma blomquisti* (Acari: Histiostomatidae). *Mitochondrial DNA Part B* **1**, 671-673 (2016).

18. Klimov, P. B. & OConnor, B. M. Improved tRNA prediction in the American house dust mite reveals widespread occurrence of extremely short minimal tRNAs in acariform mites. *BMC Genomics* **10**, 598 (2009).
19. Dermauw, W., Van Leeuwen, T., Vanholme, B. & Tirry, L. The complete mitochondrial genome of the house dust mite, *Dermatophagoides pteronyssinus* (Trouessart): a novel gene arrangement among arthropods. *BMC Genomics* **10** (2009).
20. Rider, S. D., Morgan, M. S. & Arlian, L. G. Draft genome of the scabies mite. *Parasites & vectors* **8**, 585 (2015).
21. Domes, K., Maraun, M., Scheu, S. & Cameron, S. L. The complete mitochondrial genome of the sexual oribatid mite *Steganacarus magnus*: genome rearrangements and loss of tRNAs. *BMC Genomics* **9** (2008).
22. Gu, X. *et al*. The complete mitochondrial genome of the scab mite *Psoroptes cuniculi* (Arthropoda: Arachnida) provides insights into Acari phylogeny. *Parasites & Vectors* **7**, 340-340 (2014).

**Table S6.** Partition schemes used in the present study.

| Dataset | number of partitions | Analysis method | Model and partition scheme |
|---|---|---|---|
| Nucleotide sequences | 3 | BI | GTR+I+G (ATP6_1st,, ATP8_1st,, COI_1st,, COII_1st,, COIII_1st,, CYTB_1st,, ND1_1st,, ND2_1st,, ND3_1st,, ND4_1st,, ND4L_1st,, ND5_1st,, ND6_1st) (ATP6_2nd, ATP8_2nd, COI_2nd, COII_2nd, COIII_2nd, CYTB_2nd, ND1_2nd, ND2_2nd, ND3_2nd, ND4_2nd, ND4L_2nd, ND5_2nd, ND6_2nd) (ATP6_3rd, ATP8_3rd, COI_3rd, COII_3rd, COIII_3rd, CYTB_3rd, ND1_3rd, ND2_3rd, ND3_3rd, ND4_3rd, ND4L_3rd, ND5_3rd, ND6_3rd) |
| | | ML | GTRGAMMAI (ATP6_1st,, ATP8_1st,, COI_1st,, COII_1st,, COIII_1st,, CYTB_1st,, ND1_1st,, ND2_1st,, ND3_1st,, ND4_1st,, ND4L_1st,, ND5_1st,, ND6_1st) (ATP6_2nd, ATP8_2nd, COI_2nd, COII_2nd, COIII_2nd, CYTB_2nd, ND1_2nd, ND2_2nd, ND3_2nd, ND4_2nd, ND4L_2nd, ND5_2nd, ND6_2nd) (ATP6_3rd, ATP8_3rd, COI_3rd, COII_3rd, COIII_3rd, CYTB_3rd, ND1_3rd, ND2_3rd, ND3_3rd, ND4_3rd, ND4L_3rd, ND5_3rd, ND6_3rd) |
| Amino acid | 1 | BI | WAG* (ATP6_1st, ATP6_2nd, ATP6_3rd, ATP8_1st, ATP8_2nd, ATP8_3rd, COI_1st, COI_2nd, COI_3rd, COII_1st, COII_2nd, COII_3rd, COIII_1st, COIII_2nd, COIII_3rd, CYTB_1st, CYTB_2nd, CYTB_3rd, ND1_1st, ND1_2nd, ND1_3rd, ND2_1st, ND2_2nd, ND2_3rd, ND3_1st, ND3_2nd, ND3_3rd, ND4_1st, ND4_2nd, ND4_3rd, ND4L_1st, ND4L_2nd, ND4L_3rd, ND5_1st, ND5_2nd, ND5_3rd, ND6_1st, ND6_2nd, ND6_3rd) |
| | | ML | MTARTF (ATP6_1st, ATP6_2nd, ATP6_3rd, ATP8_1st, ATP8_2nd, ATP8_3rd, COI_1st, COI_2nd, COI_3rd, COII_1st, COII_2nd, COII_3rd, COIII_1st, COIII_2nd, COIII_3rd, CYTB_1st, CYTB_2nd, CYTB_3rd, ND1_1st, ND1_2nd, ND1_3rd, ND2_1st, ND2_2nd, ND2_3rd, ND3_1st, ND3_2nd, ND3_3rd, ND4_1st, ND4_2nd, ND4_3rd, ND4L_1st, ND4L_2nd, ND4L_3rd, ND5_1st, ND5_2nd, ND5_3rd, ND6_1st, ND6_2nd, ND6_3rd) |

* MTART+I+G+F model not available in MrBayes

# Supplementary Figures

**Figure S1.** Secondary structures of tRNAs of *P. leontonychus* predicted by ARWEN, MITOS, MITOS2 and tRNAScan-SE. The tRNA short name, the source of the prediction and the calculated unconstrained and constrained MFE is indicated.

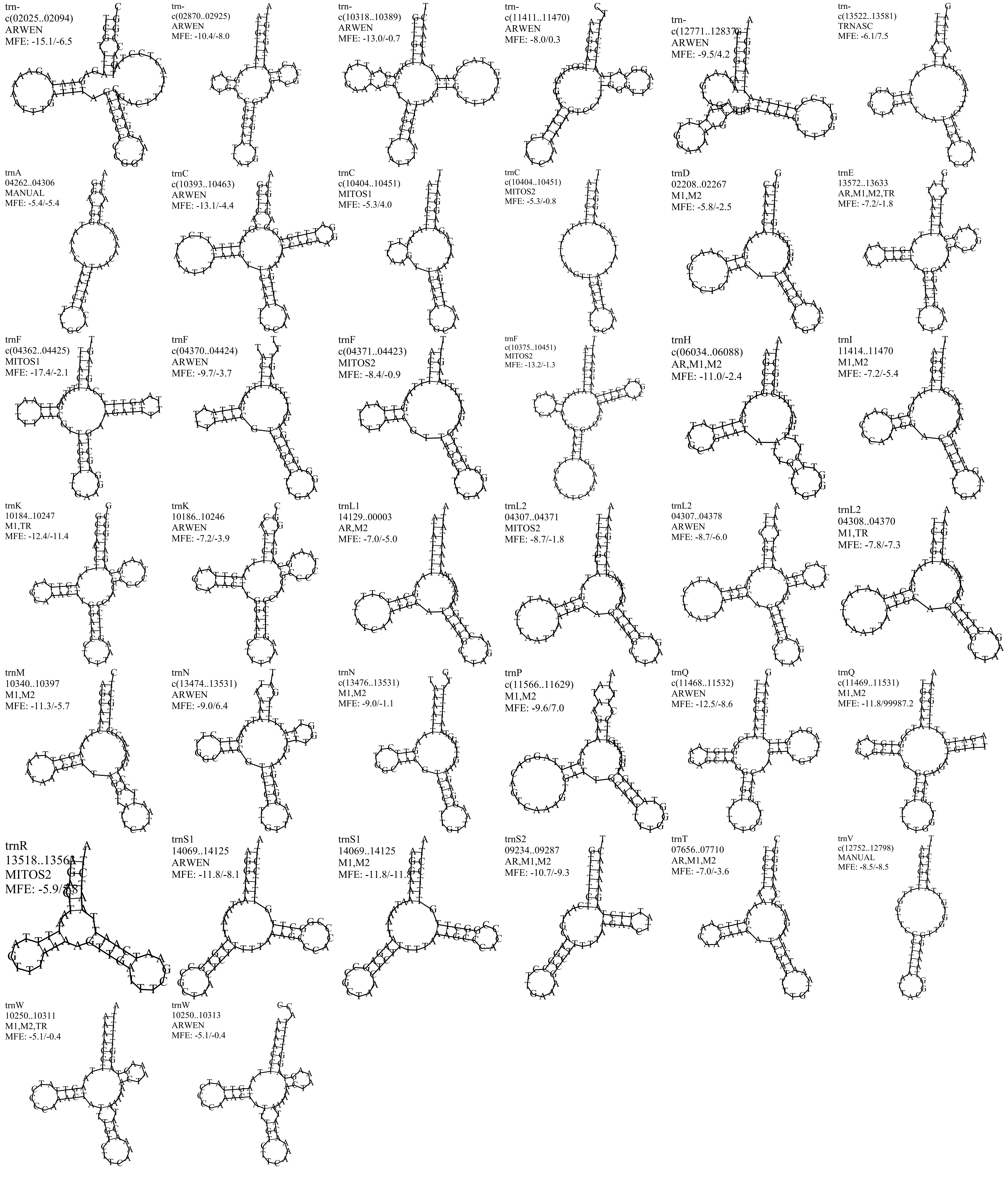

**Figure S2.** Secondary structures of tRNAs of *S. magnus* predicted by ARWEN, MITOS, MITOS2 and tRNAScan-SE. The tRNA short name, the source of the prediction and the calculated unconstrained and constrained MFE is indicated.

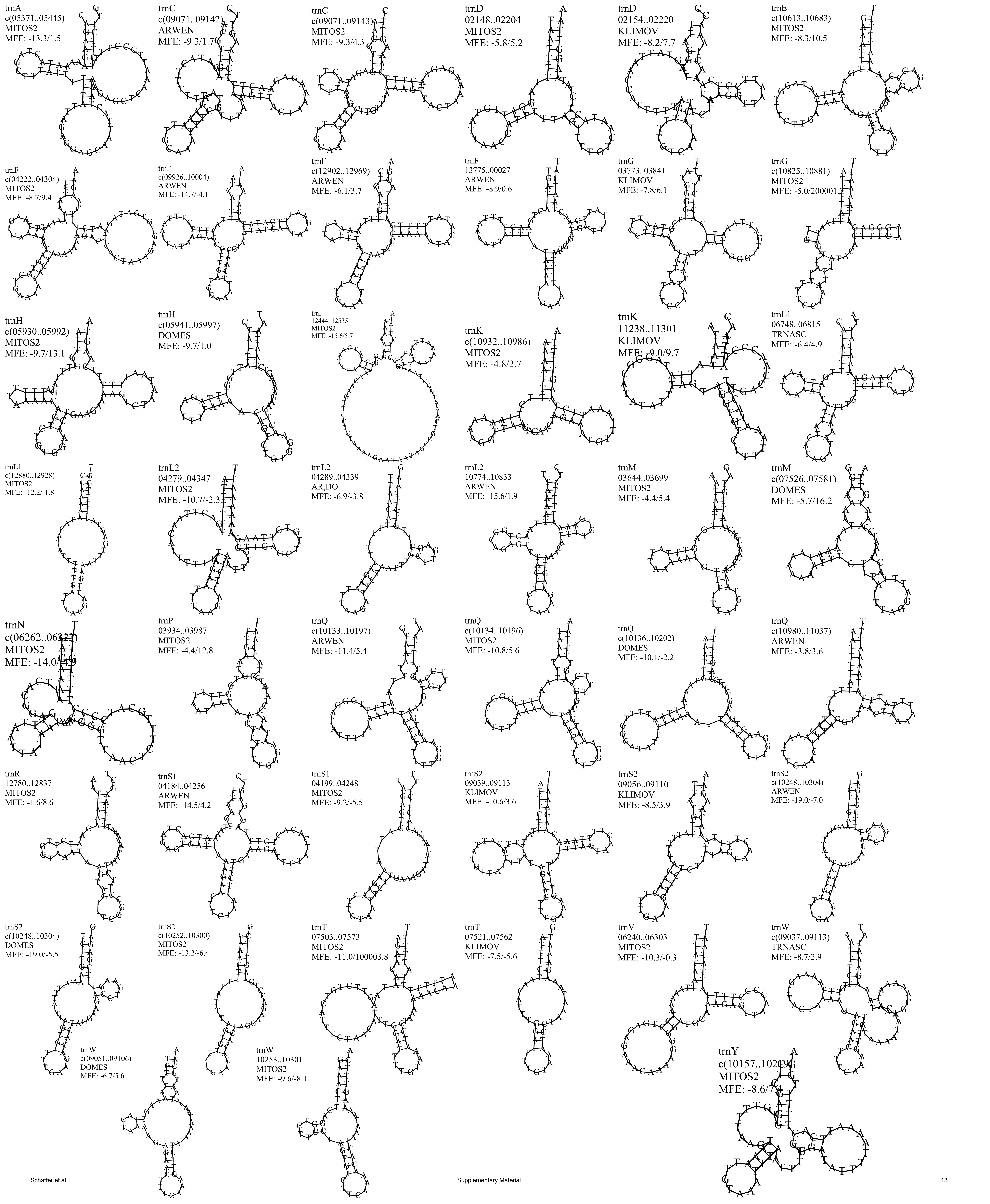

**Figure S3.** Unconstrained and Phylogenetic relationships among 37 mite taxa inferred from Bayesian Inference analyses of nucleotide (left tree) and amino acid (right tree) sequences. Numbers at nodes indicate posterior probabilities and ML bootstrap values. Acariform orders are highlighted in different colors: Astimata in light green, Oribatida in light blue and Prostigmata in mauve.

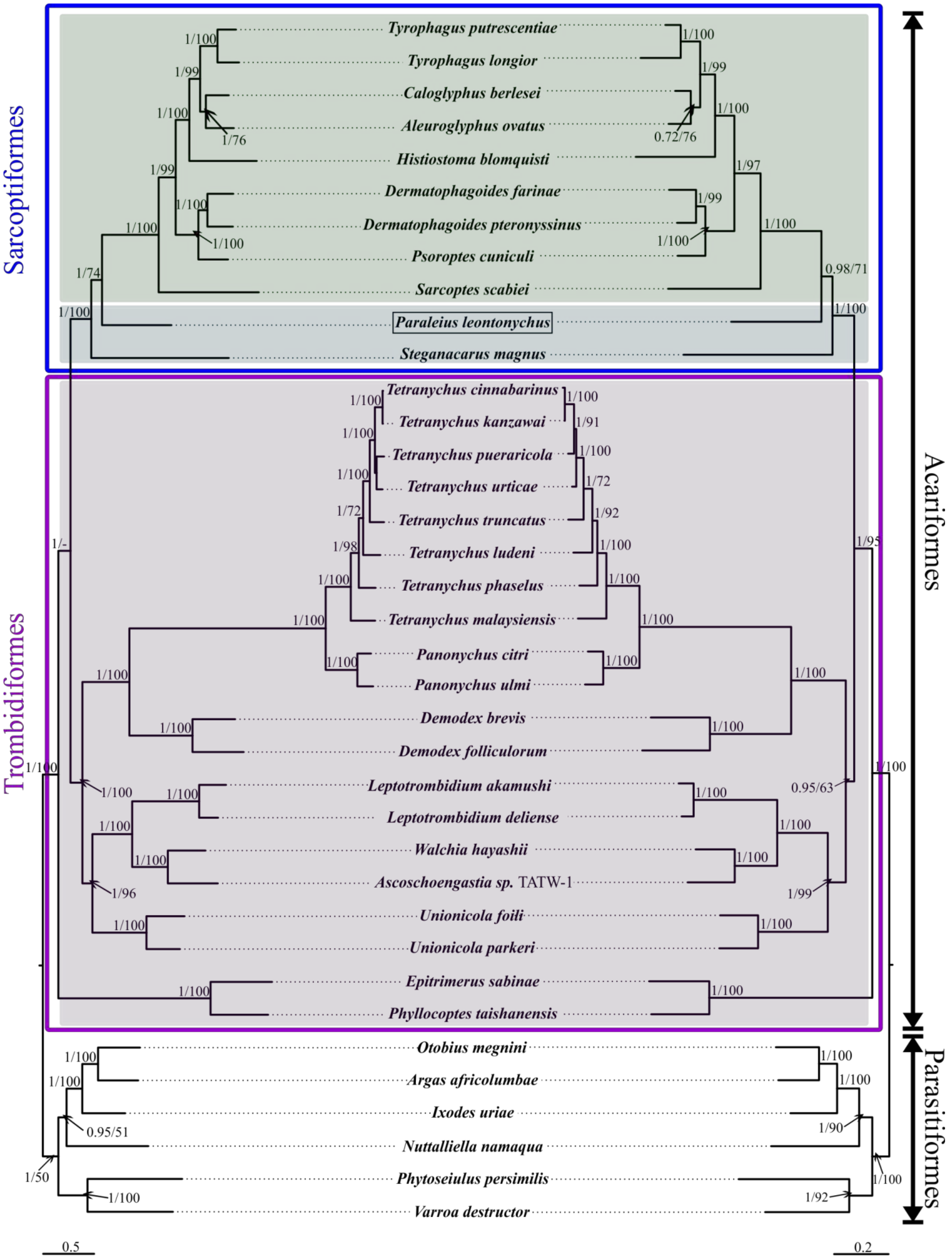

Supplement: Supplementary file 1 — Supplementary Information [file 41598_2018_25981_MOESM1_ESM.pdf]
